# An investigative study on the zoonotic potential of *Helicobacter pylori*

**DOI:** 10.1186/s12917-023-03572-w

**Published:** 2023-01-21

**Authors:** Sabah I. Shaaban, Dalia Talat, Shymaa A. Khatab, Mohamed A. Nossair, Mousa A. Ayoub, Rania M. Ewida, Mohamed Said Diab

**Affiliations:** 1 Department of Animal Hygiene and Zoonoses, Faculty of Veterinary Medicine, Damanhour University, Damanhour, 22511 Egypt; 2 Department of Microbiology, Faculty of Veterinary Medicine, Damanhour University, Damanhour, Egypt; 3 Genetics and Genetic Engineering. Department of Animal Husbandry and Animal Wealth Development, Faculty of Veterinary Medicine, Alexandria University, Alexandria, Egypt; 4 Department of Animal Hygiene and Zoonoses, Faculty of Veterinary Medicine, Alexandria University, Alexandria, Egypt; 5 Department of Food Hygiene (Milk Hygiene), Faculty of Veterinary Medicine, New Valley University, Kharga Oasis, Egypt; 6 Department of Animal Hygiene and Zoonoses, Faculty of Veterinary Medicine, New Valley University, Kharga Oasis, Egypt

**Keywords:** Animal, *glmM* gene, *H. pylori*, Human, Milk, Zoonosis

## Abstract

**Background:**

*Helicobacter pylori* is one of the most common bacterial infections and is widespread globally. It causes a variety of gastrointestinal disorders, though a great proportion of infections are asymptomatic. A total of 143 fresh stool samples were collected from apparently healthy farm and pet animals (43 cattle, 50 buffaloes, 50 sheep, 50 dogs, and 50 cats), in addition to 768 human stool samples. The samples were examined using stool antigen and rapid antibody tests, and further confirmation of *glmM* “human antigen-positive samples and animal milk samples” was conducted by polymerase chain reaction (PCR).

**Results:**

The prevalence rates of *H. pylori* infection in animals were 22.2% and 16% in antibody and stool antigen tests, respectively. The detection rates were 28%, 24%, 12%, 10%, and 4.7% in cats, dogs, buffaloes, sheep, and cattle, respectively. On the other hand, the prevalence rate of *H. pylori* infection in human stool samples was 74.8%, and a statistically significant association was observed between prevalence and several factors, such as sex, age, and locality. PCR was performed to detect the *glmM* gene of *H. pylori*, and this gene was found in 21 of 27 human antigen-positive samples and 5 of 13 animal milk samples*.*

**Conclusions:**

*H. pylori* was detected in both human and animal samples. Furthermore, *glmM* was found in milk and human samples. Our findings suggest that pet and farm animals could transmit *H. pylori* infection to humans.

## Background

*H. pylori* infection is a major public health concern in both developed and developing countries [1]. *H. pylori* is a microaerophilic gram-negative helix bacterium that specifically infects the stomach lining. The clinical manifestations are associated with the development of chronic gastritis as well as gastric and duodenal ulcers and carcinoma. However, a large proportion of infections are asymptomatic [2, 3]. The severity of clinical manifestations varies depending on several factors, such as host genetic susceptibility, the immune system, the bacterial load, and virulence [4].

The prevalence of *H. pylori* differs among societies and geographical areas and is controlled by many factors, e.g., ecological factors, sociodemographic features, population lifestyle, and hygienic practices [5, 6]. The prevalence rate of *H. pylori* is estimated to be approximately 20%–50% in developed countries, reaching 90% in Africa [7], whereas in Egypt, it is reported to be 60%–80% in the adult population [2, 8]. This global increase in prevalence may be attributed to efforts to increase the productivity of food derived from animals to meet the steadily growing population, disregarding hygienic requirements, either for the herd or personnel [9, 10].

*H. pylori* colonizes the gut of both humans and animals [11, 12]. ‏In former studies, *H. pylori* was isolated from the milk of different farming animals, such as cows, camels, ewes, and sows [13, 14]. Previous studies have also documented isolation of *H. pylori* from domestic cats [15]. Professionals handling animals and food of animal origin (e.g., veterinarians, butchers, and slaughterhouse workers) exhibit high titers of *H. pylori* antibodies (Ab). This suggests that ruminants may be the source of infection in humans [9, 16, 17].

Despite the availability of several diagnostic techniques, only tests with specificity and sensitivity beyond 90% are recommended for clinical diagnosis. *H. pylori* stool antigen tests are rapid and accurate noninvasive techniques with high sensitivity and specificity for diagnosis of active and recent *H. pylori* infection [18, 19]. Moreover, anti-*H. pylori* antibody test detection has a specificity and sensitivity of 79–90% and 76–84%, respectively [20].

Molecular techniques represent highly sensitive methods for *H. pylori* detection; indeed, they can directly detect the organism in clinical specimens. The targets of polymerase chain reaction (PCR) include *glmM*, which is the most significant indicator of *H. pylori* infection in milk and stool [9, 21, 22]. The protein encoded by *H. pylori glmM* directly participates in cell wall synthesis and plays an important and unique role in the growth and survival of the organism [23, 24].

This study aimed to investigate the prevalence of *H. pylori* infection in humans and factors associated with the infection, such as sex, age, and locality, as well as the possibility of different animal species transmitting *H. pylori* to humans.

## Results

### Detection of *H. pylori* antigen in different animal species

In this study, 39 (16%) of 243 animal stool samples tested positive when using the *H. pylori* stool antigen test. Among the 39 samples, 2, 6, 5, 12, and 14 were from cattle, buffaloes, sheep, dogs, and cats, respectively (Table 1). Statistically significant differences were observed in the prevalence of *H. pylori* infection among these animal species (*P* < 0.0001).

**Table 1:** Occurrence of *H. pylori* antigen in stool samples from apparently healthy farm and pet animals

**Species**	**No. of examined samples**	**Positive samples**	
		**No**	**%**
**Cattle**	43	2	4.7
**Buffaloes**	50	6	12.0
**Sheep**	50	5	10.0
**Dogs**	50	12	24.0
**Cats**	50	14	28.0
**Total**	243	39	16.0
**Chi**^**2**^ **value**	28.87*	*P* < 0.0001 *Statistically significant	

^*^Indicates significant differences with a *P value* < 0.0001 using the chi-squared test

### Detection of anti-*H. pylori* antibodies in different animal species

*H. pylori* antibodies were detected in 54 (22.2%) of 243 animal serum samples tested for infection using the *H. pylori* rapid antibody test. The antibodies were found to be more prevalent in pet animals (cats, 28%; dogs, 24%) than in farm animals (buffaloes, 12%; sheep, 10%; cattle, 4.7%) (Table 2). We observed a higher prevalence for the antibody test (22.2%) than the antigen test (16%) in animals, with the antibody test detecting 15 samples that were negative for *H. pylori* using the antigen test.

**Table 2:** Prevalence of *H. pylori* antibodies in different animals using the *H. pylori* rapid antibody test

**Species**	**No. of serum samples**	**Positive serum samples**	
		**No**	**%**
**Cattle**	43	8	18.6
**Buffaloes**	50	10	20.0
**Sheep**	50	8	16.0
**Dogs**	50	15	30.0
**Cats**	50	13	26.0
**Total**	243	54	22.2
**Chi**^**2**^ **value**	8.27*	*P* < 0.01	

^*^Indicates significant differences with a *P value* < 0.01 using the chi-squared test

### Prevalence of *H. pylori* in human stool samples

In this study, 575 (74.8%) of 768 human stool samples were positive for the *H. pylori* antigen. Furthermore, a statistically significant association was observed between the prevalence of *H. pylori* infection and several risk factors, such as sex, age, and locality (*P* < 0.0001) (Table 3). With respect to sex, females had a higher detection rate (79.7%) than males (65. 5%). Additionally, the occurrence of *H. pylori* increased with age: the age group > 50 years exhibited the highest prevalence (87.3%), followed by the age group 15–50 years (76.8%) and finally the age group < 15 years (16.1%). *H. pylori* was also more frequently detected in individuals living in rural areas (88.5%) than in those living in urban areas (51.7%). Therefore, *H. pylori* infection was considered a dependent variable.

**Table 3:** Prevalence of *H. pylori* infection in humans in relation to sex, age, and locality

**Variables**	**No. of examined samples**	**No. of positive samples (%)**	**Chi**^**2**^** value**	***P value***
**Sex**				
Male	264	173 (65.5)	18.65*	*P* < 0.0001
Female	504	402 (79.7)		
**Age group (years)**				
< 15	93	15 (16.1)	200.4*	*P* < 0.0001
15 and ≤ 50	302	232 (76.8)		
> 50	372	325 (87.3)		
**Locality**				
Urban	288	149 (51.7)	129.15*	*P* < 0.0001
Rural	480	425 (88.5)		

*Indicates significant differences with a *P value* < 0.01 using the chi-squared test

### Molecular detection of the *glmM* gene

**Fig. 1:**
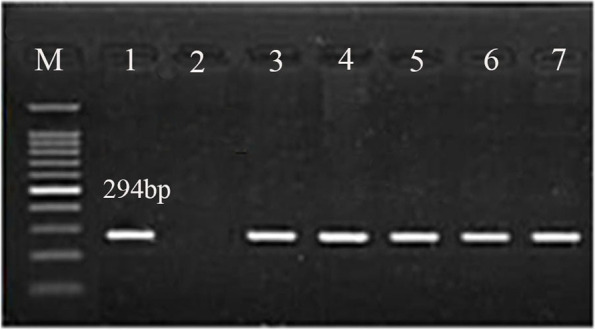
Agarose gel electrophoresis of PCR products of *glmM* (294 bp) for characterization of *H. pylori* in animal milk and human stool samples**.** Lane M: 100-bp ladder, Lane 1: control positive, Lane 2: control negative, Lanes 3 and 4: positive milk samples, Lanes 5–7 positive human stool samples

A total of 13 milk samples from farm animals with a positive *H. pylori* stool test and 27 positive human stool samples were subjected to *H. pylori glmM* detection via PCR. g*lmM* was detected in 5 and 21 milk and stool samples, respectively (Table 4 and Fig. 1).

**Table 4:** Detection frequency of *glmM* specific for *H. pylori* in milk samples and human stool samples

**Species**	**No. of examined samples**	**Positive samples**	
		**No**	**%**
**Cattle**	2	1	50.0
**Buffaloes**	6	2	33.3
**Sheep**	5	2	40.0
**Total**	13	5	38.4
**Human**	27	21	**77.8**

## Discussion

*H. pylori* infects nearly half of the population at an early age [25]. Food, particularly milk, is one of the most important potential sources of infection in humans [9, 14].

Regarding animal stool samples, the *H. pylori* antigen (Table 1) was most frequently detected in cats (28%) in our study, followed by dogs (24%), buffaloes (12%), sheep (10%), and cattle (4.7%). In Iran, detection frequencies were reportedly 3% and 16% in cows and sheep, respectively [17]. In Italy, a higher prevalence was recorded in dog and sheep stool samples, at 100% and 82%, respectively [26].

In the sera of diverse animals (Table 2), *H. pylori* antibodies were most frequently detected in dogs (30%), followed by cats (26%), buffaloes (20%), cattle (18.6%), and sheep (16%), with a statistically significant association between these frequency rates. These rates were in agreement with those in cow sera (27%) in Iran [27]; detection rate in Algerian local-breed cows is 12% [9]. In contrast, higher detection rates have been detected in sheep (68%), cattle (70.3%), and goats (96.4%) in Sudan [28]. Additionally, *H. pylori* was found in 100% of dogs and 95% of sheep in northern Sardinia [26]. Overall, the antibody test results in our study were higher than those of the rapid Ag test, which could be explained by the fact that Ab seropositivity persists for a longer period of time, possibly up to 3 years [29].

Regarding human stool samples, the overall detection rate of *H. pylori* was 74.8% using the rapid antigen test. Nearly similar results were recorded among Egyptian patients with dyspepsia (73.7%) [2]. Furthermore, in west Iran, the *H. pylori* antigen positivity was 64.2% [30]. However, these results were higher than those reported in Japan (56.4%) [31], Ethiopia (49.7%) [32], and Khuzestan province, Iran (53.5%) [33]. In addition, our results were much higher than those reported in Turkey (36.6%) [34] and India (42.8%) [35], though the results in Pakistan (25%) [36] and Ethiopia (30.4%) were lower [37]. This significant variation in frequency might be due to the use of various techniques for *H. pylori* detection, socioeconomic status of the studied population, geographical variations, and environmental changes due to poor control measures, leading to exposure to raw sewage particles.

The association between the prevalence of *H. pylori* infection in humans and the sex of participants is illustrated in Table 3, with the infection rate in females (79.7%) and males (65.5%) showing a statistically significant association (*P* < 0.0001). The same finding was obtained in studies conducted in Oman [38] and Egypt [2]. This could be explained by the fact that women are more caring about their health than are men and often seek treatment for health problems. However, in a study on Australian patients, *H*. *pylori* infection rates were higher in males than in females [39]. This could be attributed to the fact that males tend to work outside of the home for longer periods of time than do women; thus, they are more exposed to environmental factors. In Ethiopia [37] and Sudan [40], no relationship between *H. pylori* infection and patient sex was observed.

Statistical analysis of the data presented in Table 3 revealed a significant difference (*P* < 0.0001) between various age groups and *H. pylori* prevalence. The highest prevalence was observed in the age group > 50 years (87.3%), followed by the age group 15–50 years (76.8%) and the age group < 15 years (16.1%). However, our study disagrees with previous studies in Egypt [2] and Sudan [40]. In addition, no statistically significant association was observed between the prevalence of *H. pylori* infection and age in Ethiopia [37]. These contradictory findings may be due to differences in the studied population and sample size.

The effect of participants' habitat on the prevalence of *H. pylori* infection revealed a statistically significant association (*P* < 0.0001). The infection was prevalent in those living in rural areas, where "there is an increased likelihood of contact with animals" (88.5%), and in urban areas (51.7%) (Table 3). Our findings were in agreement with those of a study conducted in Egypt reporting that farmers are more prone to *H. pylori* infection (82.1%) than non-farmers (64.9%) [2]. The higher prevalence of infection among rural inhabitants supports the theory that such an infection is a zoonosis. This was proposed by some previous studies in Egypt that isolated the studied organism from stool and milk samples of cattle [13]. Furthermore, a study in Iran confirmed isolation of *H. pylori* from gastric samples of several animals, such as sheep and cows, and considered them potential sources of *H. pylori* infection in humans [17]. In addition, the higher prevalence of *H. pylori* among rural inhabitants in our study may be attributed to the conditions that promote transmission, such as lack of income, poor hygiene, and low educational level. These results indicate that farm animals may be sources of *H. pylori* infection among humans, especially those who are handling them or consuming their products, such as milk.

g*lmM* is important for cell wall development and microorganism growth. It has also been used extensively for molecular confirmation of *H. pylori* owing to its high specificity [41].

Milk is often consumed by humans, especially children. Thus, consuming milk represents the most proposed theory for *H. pylori* transmission from animals to humans. *H. pylori* has been detected in both pasteurized milk and raw milk in addition to the milk products of goats, sheep, and cows using conventional methods, molecular techniques, or both [42, 43]. Regarding milk, 13 samples were examined for the presence of *H. pylori glmM* in our study, with detection in five (38.4%) samples (one from cattle, two from buffaloes, and two from sheep). This finding was supported by former studies in Italy [43] and Sudan, which detected the presence of *glmM* in 22% of milk samples examined [44]. On the other hand, lower rates were recorded in Iran, including 8.7% in goat, 12.2% in sheep, and 14.1% in cow milk samples [45]. Furthermore, 13% of the Algerian local-breed cows were *glmM* positive, representing a possible zoonosis [9]. The presence of *H. pylori* in milk indicates the increasing hazard of human infection via consumption of contaminated raw milk [39]. Therefore, food safety and quality standards as well as good dairy farming practices are required for the production of milk that is safe for human consumption.

In this study, the prevalence of *glmM* in human stool samples was 77.8%. This finding is consistent with a study conducted in Iran that confirmed the existence of *glmM* in antigen-positive stool samples [27] and a study in Egypt that confirmed the presence of *glmM* in 64.7% of *H. pylori*-infected stool samples from sewage workers [46]. A slightly higher prevalence (93.7%) was observed in stool samples obtained from patients with gastritis [47]. In Iran, lower levels of *glmM* (39%) were found in stool samples from pediatric patients [48].

## Conclusion

In El-Beheira Governorate, Egypt, *H. pylori* is frequently detected in both humans and animals. Factors such as sex, age, and locality were found to be associated with *H. pylori* infection in humans. *H. pylori* was more frequently detected in pet animals than in farm animals, and milk is a possible source of transmission to humans. However, further studies are warranted to provide more information regarding the exact sources of infection and the role of animals in disease transmission. *H. pylori* poses a great public hazard; therefore, we recommend that public health authorities prioritize the development of preventive measures against *H. pylori* infection.

## Methods

### Ethical declaration

This research was conducted according to guidelines of the Institutional Animal Care and Use Committee of Alexandria University (ALEXU-IACUC, 3312020) in Egypt. Informed consent was obtained from the human participants and/or their legal guardian after receiving detailed information about the aims of the study.

### Study area and design

The present study was conducted from January to December 2021 at El-Beheira Governorate, Egypt, to determine the prevalence of *H. pylori* in different animal species and humans.

### Sampling

#### Animal samples

A total of 143 samples were obtained from apparently healthy farm animals, namely, cattle (*n* = 43), buffaloes (*n* = 50), and sheep (*n* = 50). The animals were Egyptian native breeds and reared in a semi-intensive system. Samples, such as stool, serum, and milk, were collected from each animal investigated. Stool samples were collected in sterile dry, disposable, leak-proof, wide-mouthed, plastic containers, and 5–7-mL blood samples were collected in plain Vacutainer tubes and kept at room temperature for 1–2 h before storage; raw milk samples were placed in sterile Falcon tubes. Furthermore, 100 stool samples were randomly collected from pet animals in veterinary clinics, including dogs and cats (50 samples each). All the collected samples were labeled and immediately transferred to the laboratory in an icebox for examination.

#### Human samples

A total of 768 fresh stool samples were collected from patients with dyspepsia (264 males and 504 females) aged 5–80 years who visited the Gastroenterology Department in hospitals in El-Beheira Governorate, Egypt. Personal and sociodemographic data relevant to risk factors for infection were collected, such as sex, age, and locality. The patients were divided into three age groups (< 15, 15–50, and > 50 years). The samples were collected in sterile airtight cups, labeled, and then immediately transferred to the laboratory in an icebox.

### *H. pylori* antigen detection

Stool samples (from animals and humans) were examined for the presence of *H. pylori*-specific antigen using *H. pylori* Ag Rapid Test-Cassette (OnSite®, CTK Biotech, San Diego, USA), a lateral-flow chromatographic immunoassay for the qualitative detection of *H. pylori* antigen in stool samples [49, 50]. The procedures and reading of the results were performed according to the manufacturer’s protocols.

### *H. pylori* rapid antibody test (serological examination)

An antibody rapid test device (serum/plasma) (Atlas Medical, Cambridge, England, and Model No. 8.04.21) was used as a qualitative membrane-based immunoassay to investigate the presence of IgM and IgG antibodies specific for *H. pylori* in serum samples [51]. The procedures and interpretation of the results were performed according to the manufacturer’s protocols.

### Detection of *H. pylori glmM *via PCR

DNA was extracted from milk samples using a DNA isolation kit (Cat. No. ABIN412492, Roche, Mannhein, Germany) according to the manufacturer’s protocols. Furthermore, a QIAamp DNA Stool Mini Kit (QIAGEN, Hilden, Germany) was used to extract DNA from stool samples according to the manufacturer’s protocols. Specific oligonucleotide primers (Table 5) were used to amplify a 294-bp region of the *H. pylori* *glmM* gene [45]. PCR for *glmM* was performed under the following conditions: initial denaturation at 94 °C for 10 min, 35 cycles of denaturation at 94 °C for 60 s, annealing at 55 °C for 60 s, extension at 72 °C for 60 s, and a final extension at 72 °C for 10 min. The PCR products were subjected to 1.5% agarose gel electrophoresis (Bio-Rad, France) with ethidium bromide staining.

**Table 5:** Oligonucleotide primer sequences used for the detection of *glmM*

**Target gene**	**Oligonucleotide sequence 5′–3′**	**Size (bp)**
*glmM*	GAATAAGCTTTTAGGGGTGTTAGGGG GCTTACTTTCTAACACTAACGCGC	294

### Statistical analysis

Statistical analysis was conducted using the chi-squared (Chi^2^) test to examine significant differences between the studied groups according to SAS (2014). *P* values < 0.05 were considered to indicate significant differences.

## Data Availability

The datasets used and/or analyzed in the current study were not publicly published to preserve the privacy of the participants but are available upon reasonable request from the corresponding author.
